# Virtual pivot point: Always experimentally observed in human walking?

**DOI:** 10.1371/journal.pone.0292874

**Published:** 2023-10-13

**Authors:** Johanna Vielemeyer, Lucas Schreff, Stefan Hochstein, Roy Müller

**Affiliations:** 1 Institute of Sport Sciences, Friedrich-Schiller-University Jena, Jena, Germany; 2 GaitLab, Klinikum Bayreuth GmbH, Bayreuth, Germany; 3 Bayreuth Center of Sport Science, University of Bayreuth, Bayreuth, Germany; 4 Institute of Sport Sciences, Martin-Luther-University Halle-Wittenberg, Halle, Germany; Polytechnic University of Marche: Universita Politecnica delle Marche, ITALY

## Abstract

A main challenge in human walking is maintaining stability. One strategy to balance the whole body dynamically is to direct the ground reaction forces toward a point above the center of mass, called virtual pivot point (VPP). This strategy could be observed in various experimental studies for human and animal gait. A VPP was also observed when VPP input variables like center of mass or ground reaction forces were perturbed. In this study, the kinetic and kinematic consequences of a center of pressure manipulation and the influence on the VPP are investigated. Thus, eleven participants walked with manipulated center of pressure (i.e. barefoot, backwards, with a rigid sole, with stilts, and in handstand compared to shoe walking). In all conditions a VPP could be observed, only one participant showed no VPP in handstand walking. The vertical VPP position only differs between shoe walking and rigid sole walking, there are no significant differences between the conditions in the horizontal VPP position and the spread around the VPP. However, it is conceivable that for more severe gait changes, walking without VPP could be observed. To further analyze this issue, the authors provide a VPP calculation tool for testing data regarding the existence of the VPP.

## Introduction

One of the main challenges for the human gait is maintaining stability. A mechanical strategy for stabilizing the whole body dynamically is to direct the ground reaction forces (GRFs), starting at the center of pressure (CoP), to a point above the center of mass of the whole body (CoM). This point is called virtual pivot point (VPP) [1]. Until now, in experimental studies a VPP could always be observed: for human walking [1–3], human running [4], and even in animals like dogs [1, 5], macaques [6], and quails [7].

The mechanics of the human body is complex and the constituents of the musculoskeletal system include muscles, tendons and ligaments that move the body segments. Since the interaction of the musculoskeletal system creates the VPP, the interaction of the CoM, CoP, and GRFs are of interest when analyzing gait. Thus, various studies manipulated some of those parameters to examine whether a VPP can still be found. To change the CoM position and thus the GRFs, Müller et al. [8] investigated human walking with different trunk inclinations. A similar approach, but with extrinsic perturbations instead of intrinsic changes of the body posture, was chosen by a walking [9] as well as a running [4] study. In both studies, the gait over visible and camouflaged curbs was examined to determine the influence of the perturbed CoM and GRFs on the existence and position of the VPP. In all of the above mentioned studies, a VPP could be found. Until now, however, the influence of the CoP on the VPP has not been explicitly investigated and thus is the topic of this study. Therefore, the role of the CoP while walking is briefly summarized below.

During the single support phase of human walking, the horizontal motion of the CoP consists of two components. The stepping, which is the motion of the stance foot relative to the CoM, and the foot rollover, that is the motion of the CoP relative to the foot [10]. It was found that the stepping component alone suffices to walk upright, however with less angular motion of the whole body [10]. With only stepping, the GRFs seem to be directed to the CoM. With the foot rollover, they point above the CoM [10]. To maintain balance in human walking, foot placement adjustments or modulations of joint moments (e.g. ankle, hip) could be made [11]. Thus, the CoP motion has an influence on the balance and the regulation of the angular motion. Since atypical regulation of the angular motion in human walking is associated with a risk of falling [12, 13], it is important to understand how humans regulate their angular motions to aid fall prevention research [10]. Therefore, in this study, the kinetic and kinematic consequences of CoP manipulation and the relation to the VPP are investigated. Both stepping and foot rollover will be manipulated.

To change primarily the foot rollover, barefoot walking (more flexibility in metatarsophalangeal joints), walking with rigid soles due to a plank in the shoe (to avoid movement in metatarsophalangeal joints), and walking with stilts (to additionally reduce the support area) are analyzed in this study. For the manipulation of the stepping and the rollover component, other gait types than normal upright walking like backwards and handstand walking are investigated here. Both gaits were attested to have substantial potential for understanding the neuromuscular control of human locomotive behavior [14, 15].

A VPP could also occur when walking without foot rollover, as some animals like dogs and chickens show this behavior [1, 10]. Thus, in this study it is hypothesized that (1) for walking with modified foot rollover a VPP could be observed, but the position will change. If the range of the CoP motion relative to the horizontal CoM position becomes smaller, the VPP is shifted down [16]. For walking with a plank the range will presumably be smaller than for walking with shoes since the foot lift-off will take place further ahead in the foot due to the limited rollover in the metatarsophalangeal joints. For barefoot walking as well as for stilt walking the range will become correspondingly larger, because here, the absolute CoP is considerably shortened so that it is further away from the CoM. That is, the CoP relative to the CoM is expected to be lengthened which shifts the VPP upwards. In contrast, for plank walking it is expected that the CoP relative to the CoM will be shortened which shifts the VPP downwards. Additionally, it is hypothesized that (2) the changed stepping component combined with changed rollover in backwards and handstand walking will increase the spread around the VPP because of a more challenging neuromuscular control.

## Materials and methods

### Participants

Thirteen participants (10 women, 3 men) took part in this study. Two men were excluded from the evaluation because of missing or incorrect kinematic data. The remaining eleven participants were between 18 and 31 years old (mean±std, age: 24.3±4.0 years, height: 1.65±0.09 m, mass: 57.7±6.1 kg) and were all physical active and had experience in handstand walk (could walk at least five steps without falling). They had no known limitations that could have affected their performance in the experiment.

Before the start of the experiment, an informed written consent form was signed by each participant. The study was approved by the local ethics committee (University of Jena, 3532-08/12) and performed in accordance with the Declaration of Helsinki. Participants were recruited in October 2022 just before the data collection at the Biomechanical Laboratory of the Sports Institute within Friedrich Schiller University Jena. All data were collected pseudonymously and only the first author has access to the key that could match individual participants to pseudonymized data.

### Measurements

**Fig 1:**
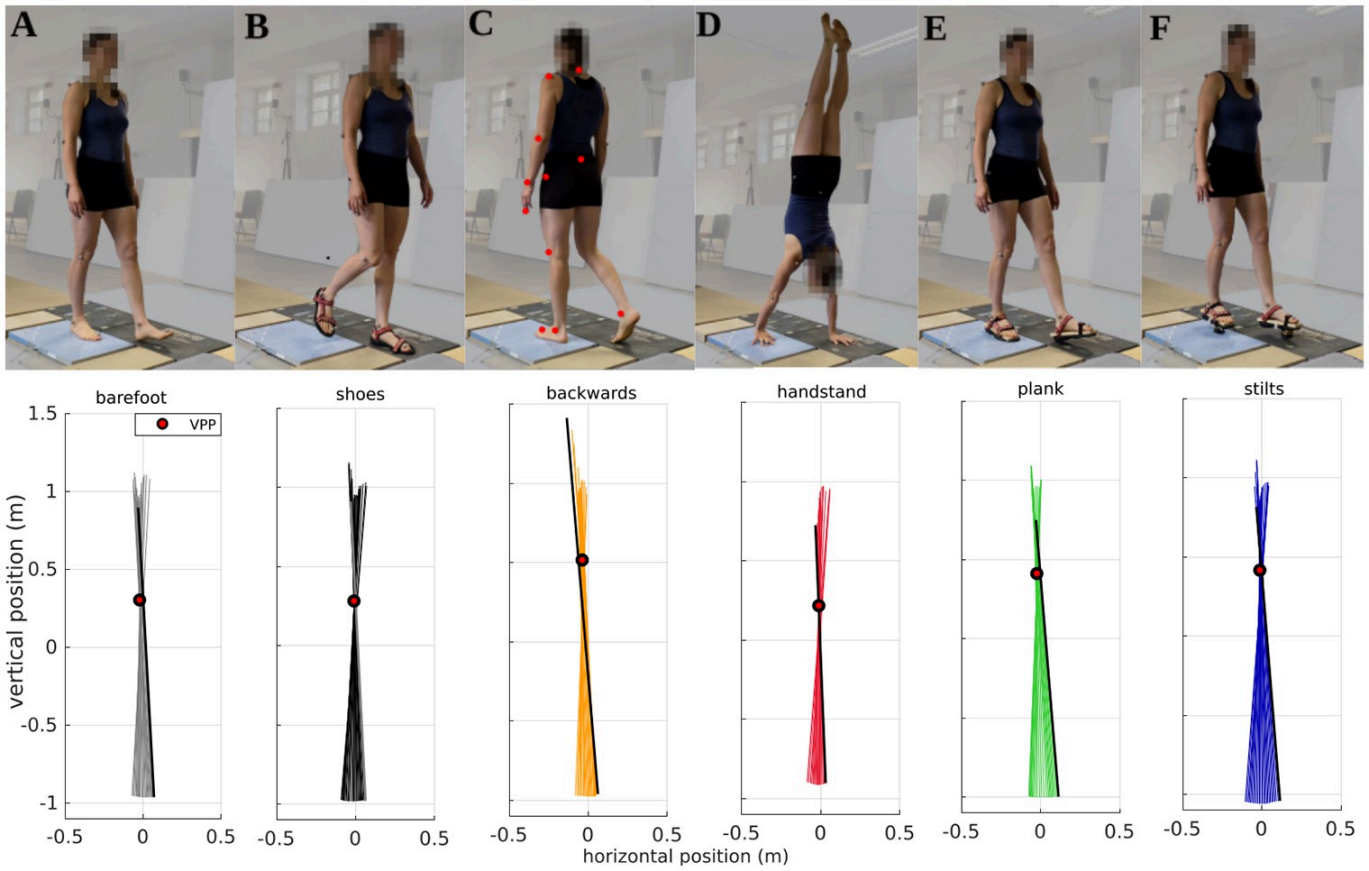
Experimental conditions and exemplary plots of the virtual pivot point (VPP) of one representative participant. Above: Walking A: barefoot, B: with shoes, C: backwards, D: in handstand, E: with planks, and F: with stilts. The placement of the reflective joint markers of one body side is indicated by the red circles in C. Photo credit: Sandro Schwarzentrub. Below: Colored lines show the ground reaction forces (GRFs) at different measurement times originating at the center of pressure in a coordinate system centered on the center of mass. The GRFs are illustrated from touchdown (black/gray line) to take-off. Red circles with black borders indicate the calculated VPP. For each condition, the first trial is shown.

The participants were asked to walk in different walking styles along a 5 m walkway with three consecutive force plates in its center (Fig 1). The walking styles were barefoot, backwards, and handstand walking as well as walking with shoes, planks, and stilts. Backward and handstand walking were performed barefoot. The shoes were Teva^®^ sandals, in which a wooden plank (height: 1.5 cm, length and width: same as shoes) was put in for the plank condition (Fig 1). For the stilt condition, a wooden pin (height: 5 cm, surface area: 4.5x4.5 cm) with rubber base was attached under the shoe with the plank inside. The order of the conditions was block randomized. Because of the force plate configuration, it was predetermined with which foot or hand the force plates must be hit. To exclude side effects, the walking direction was alternated for each trial.

A metronome was used at 80 bpm to control the step cadence in all conditions, with one step on each click. Each of the three force plates (outer: 9281B11, inner: 9286BA; Kistler, Winterthur, Switzerland) had to be hit with one foot or hand. Since the positions of the force plates were adapted to the step length of handstand walking, it was allowed at the first and third force plates to overlap to the wooden track to lengthen the steps in normal walking. This was possible because these two force plates were only used to determine the exact time for the take-off (TO) before the second contact and the touchdown (TD) after the second contact, respectively. All force plates were sampled at 960 Hz. The walking track was surrounded by mats to minimize injury risk.

Several practice trials were conducted before each condition to ensure that the task had been realized appropriately. Due to the low speed, it was sufficient to perform only 1-2 preparatory steps to reach the target speed before the measured contacts. For each condition, ten trials were performed.

The experiment was recorded with seven cameras (240 Hz) by a 3D infrared system (MCU 1000, Qualisys, Gothenburg, Sweden) and synchronized using the trigger of the Kistler software and hardware. The reflective joint markers were positioned on acromion, epicondylus lateralis humeri, ulnar styloid processus, tip of the third finger, trochanter major, epicondylus lateralis femoris, lateral malleolus, medial malleolus, and tip of the first toe on both sides of the body as well as on L5 and C7 process spinosus (Fig 1C).

### Data processing

Data were analyzed with custom-written Matlab code (The Mathworks, Inc., Natick, MA, USA). Raw kinematic data were filtered at 50 Hz cutoff frequency with a bidirectional fourth-order low-pass Butterworth filter [8, 17]. Kinetic data were normalized to individual body weight (BW). The instants of TD and TO were calculated as the events at which the GRFs exceeded or fell below the threshold of 0.05 BW, respectively. The CoM was calculated using the kinematics and a body segment parameter method according to Plagenhoef et al. [18]. The masses of the shoes, stilts and planks were negligible. The step length and step width were computed as the anterior-posterior and mediolateral distances between the lateral malleolus of the left and right foot at the corresponding TDs.

The position of the VPP (in horizontal direction VPPx and in vertical direction VPPz) was calculated using GRF vectors starting at the CoP for every instant of measurement. The vectors were analyzed in a CoM-centered coordinate frame with a vertical axis parallel to gravity. The VPP position is defined as the point where the sum of the squared perpendicular distances to the GRFs in the single support phase (i.e. from TO to the following TD) is minimal. To describe whether the sum of the distances between GRFs and VPP is low or high, a coefficient of determination, named R^2^, was considered. This coefficient was used to evaluate whether the VPP can be denoted as a point or not. Additionally, with R^2^, the deviation from a point can be assessed. It was calculated by using the angle between the experimentally measured GRFs and the line between the CoP and the computed VPP. Specifically, R^2^ was calculated as follows, adapted from Herr & Popovic [19]:

$$\begin{array}{c} \begin{array}{c} R^{2}\;=\;1\;-\;\frac{\sum_{i=1}^{N_{Trial}}\;\sum_{j=1}^{N_{Time}}{\left(\theta_{\text{Exp}}^{ij}-\theta_{\text{VPP}}^{ij}\right)}^{2}}{\sum_{i=1}^{N_{Trial}}\;\sum_{j=1}^{N_{Time}}{\left(\theta_{\text{Exp}}^{ij}-{\bar{\theta }}_{\text{Exp}}\right)}^{2}} \end{array} \end{array}$$ (1)

with at least one pair of *i*, *j*, such that $\theta_{\text{Exp}}^{ij}\neq {\bar{\theta }}_{\text{Exp}}$. Here, *θ*_Exp_ is the angle between the ground and the experimentally measured GRFs and *θ*_VPP_ is the angle between the ground and the theoretical forces (i.e. the linear connections between the CoP and the computed VPP). The mean experimental angle ${\bar{\theta }}_{\text{Exp}}$ is the grand mean over all trials and measurement times. The R^2^ values were calculated for each participant and condition by summing over all trials (*N*_*Trial*_) and measurement times (*N*_*Time*_). Note that the values of R^2^ can vary between −∞ and 1 per definition and that an R^2^ value of 1 indicates a perfect fit between model and experiment and an R^2^ value of 0 or negative values mean that the estimation of the model is equal to or worse than using the mean experimental value as an estimate [19]. Based on statements of Herr & Popovic [19], here the VPP was defined as a point if R^2^ was greater than 0.6, separately for each condition. For each trial, the VPP position was only considered if the VPP was classified as a point (for more details concerning the VPP calculation see [16]). For the analyses, the anterior-posterior (x) direction and the vertical (z) direction were considered.

The (centroidal) angular momentum of the whole body was calculated as described in Herr & Popovic [19] and normalized to the body mass, the CoM height, and the mean velocity of each condition. The ankle/wrist torque was computed as in Rouse et al. [20] and normalized to the body mass of each participant.

As spatio-temporal parameters, walking speed, contact time, absolute single and double support time, single support time relative to the whole contact, step length and width and the cadence were analyzed. To compare spatio-temporal gait parameters and VPP variables between conditions, repeated measures ANOVA (P<0.05) with post-hoc analysis (Šidák correction) were used. Conditions were barefoot, shoes, backwards, handstand, plank, and stilts. To analyze whether the VPP was above, below or at the CoM, and anterior or posterior to it, t-tests compared to zero were performed (separately for each condition with Šidák correction). To describe differences between conditions, the mean value over all trials was calculated. Then, for the VPP variables, median±’median absolute deviation’ between participants was computed to reduce the weight of outliers. For all other variables, the mean±s.d. of the participants was calculated. The terms ‘anterior’ and ‘posterior’ refer to the walking direction (in walking direction means ‘anterior’).

## Results

**Fig 2:**
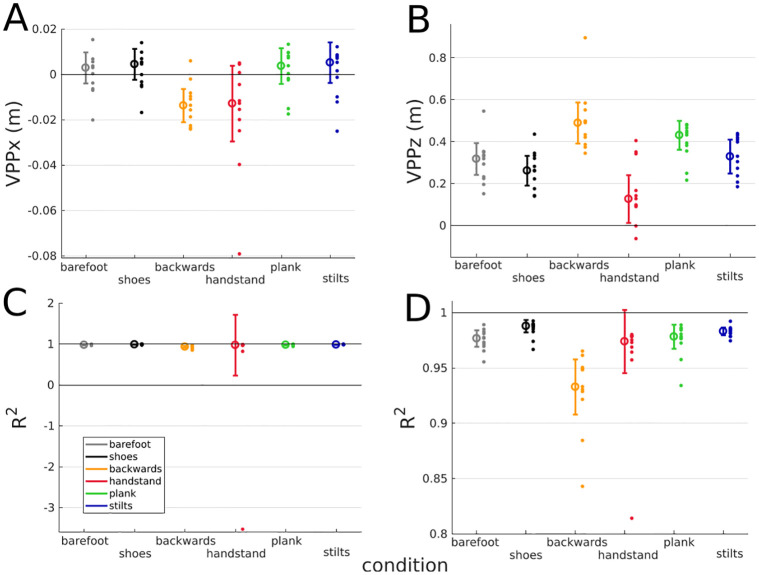
Median±’median absolute deviation’ of the virtual pivot point (VPP) variables between participants for each experimental condition. A: Horizontal (x) and B: vertical (z) position of the VPP, each small dot represents the mean over all trials of one condition for one participant. Only trials with R^2^>0.6 were considered. R^2^ represents the spread around the VPP. C: All R^2^ values are shown (N = 11), D: only participants with R^2^>0.6 are included (handstand: N = 10, else: N = 11). Each small dot represents one participant.

**Fig 3:**
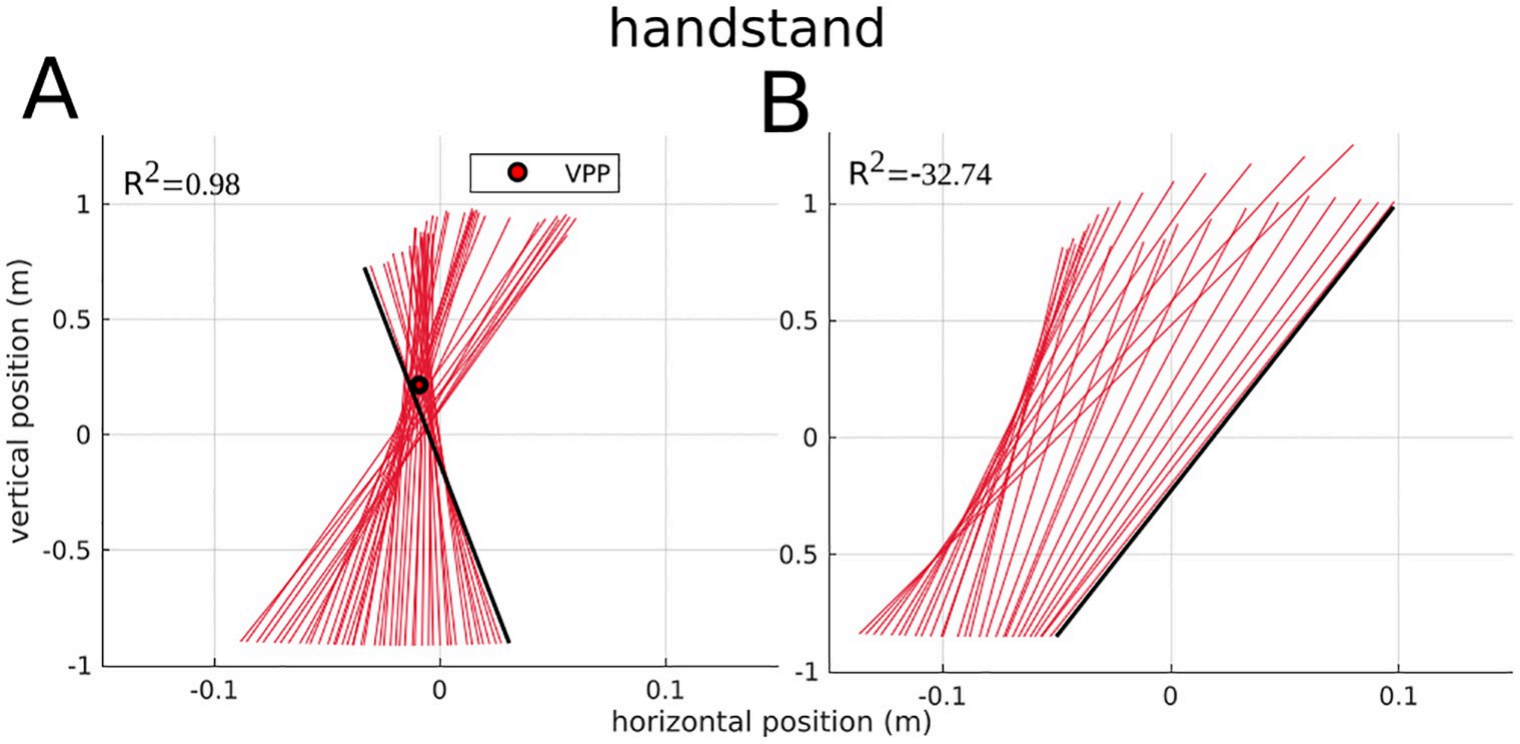
Example virtual pivot point (VPP) plots for handstand walking. A: One trial of one representative participant (compare Fig 1F) and B: one trial of the one participant with a median R^2^<0. Note, that most of the other trials for this participant at handstand walking look similar. Colored lines show the ground reaction forces (GRFs) at different measurement times originating at the center of pressure in a coordinate system centered on the center of mass. The GRFs are illustrated from touchdown (black line) to take-off. Red circle with black border indicates the calculated VPP if R^2^>0.6.

**Fig 4:**
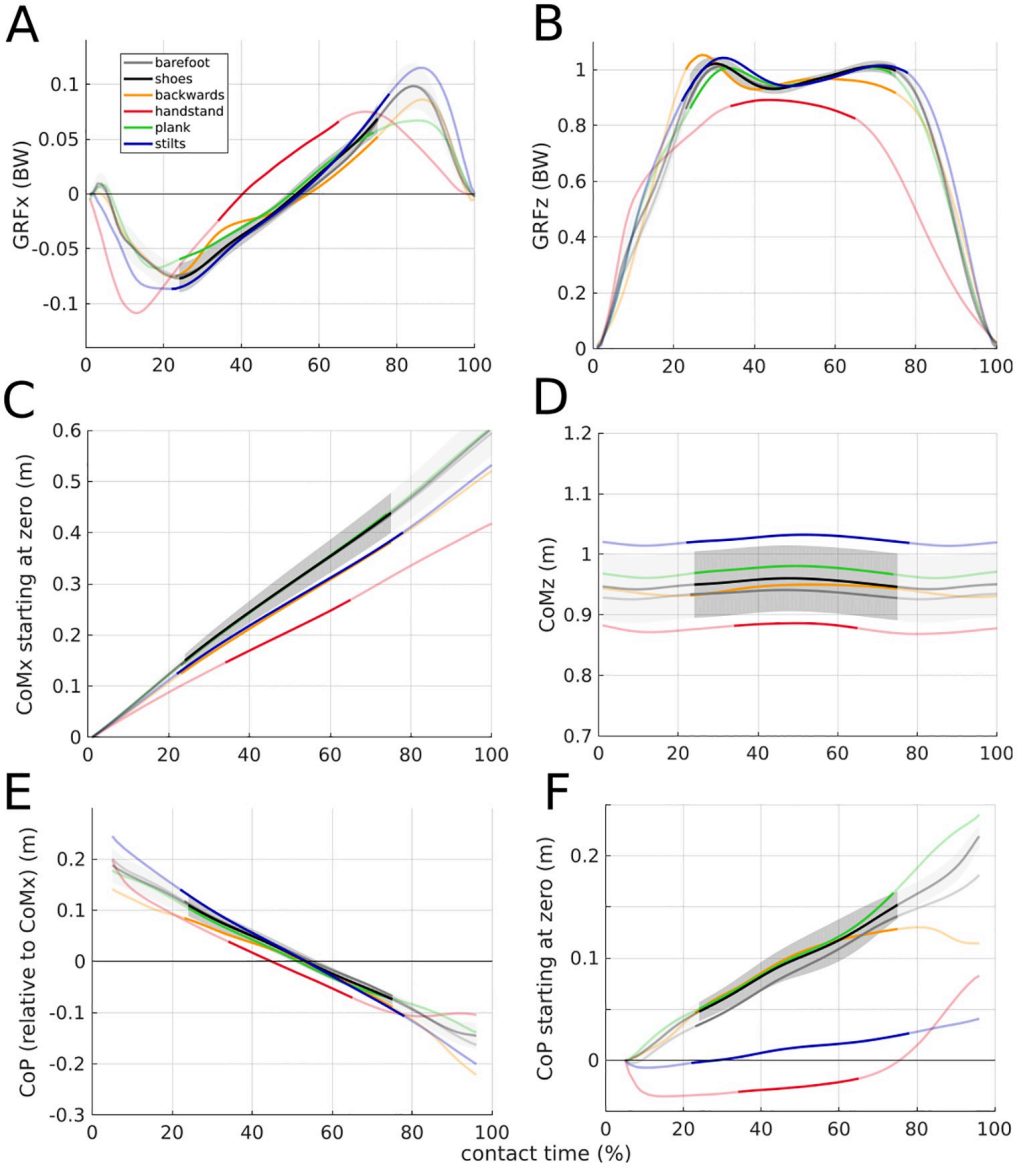
Variables included in the calculation of the virtual pivot point (VPP). All experimental conditions are shown. Values are mean of all trials and subsequent mean of all participants (N = 11). For shoe walking, mean±s.d. (gray area) is shown. The non-transparent trajectory represents the single support phase, for which the VPP was calculated. A: Horizontal (x) ground reaction forces (GRFs), B: vertical (z) GRFs proportional to body weight (BW). C: Horizontal center of mass (CoM) position normalized to zero at touchdown, D: vertical CoM position. E: Horizontal, CoM-related center of pressure (CoP) position shown for 5% to 95% of contact time due to noisy CoP at the edges. F: Horizontal CoP position normalized to zero at 5% of the contact time, shown for 5% to 95% of contact time.

**Fig 5:**
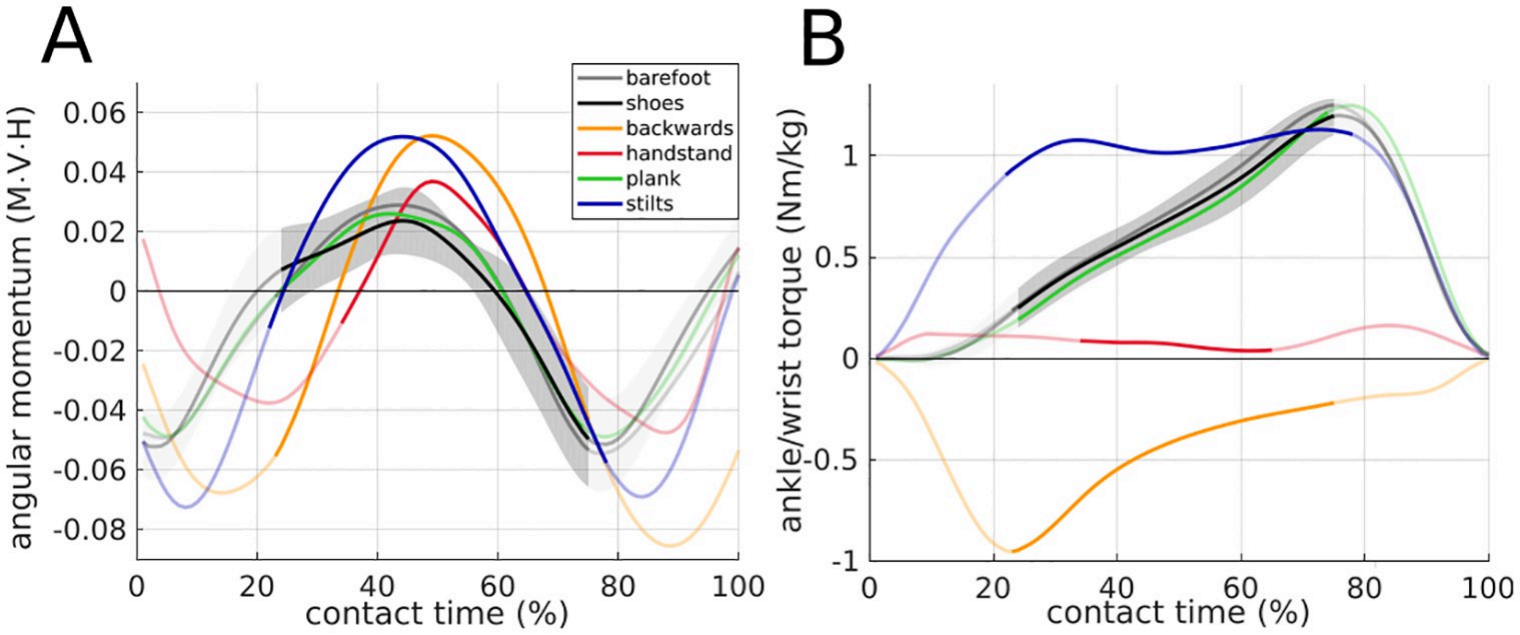
Angular momentum and ankle torque. All experimental conditions are shown. Values are mean of all trials and subsequent mean of concluded participants. For shoe walking, mean±s.d. (gray area) is shown. The non-transparent trajectory represents the single support phase, for which the VPP was calculated. A: The angular momentum was normalized to body mass (M), mean walking velocity of each condition (V), and mean center of mass height (H). Negative values indicate clockwise rotation (N = 11). B: Ankle torque (for handstand walking wrist torque) normalized to body mass is shown (handstand: N = 9, else: N = 11).

The results and statistical values are listed in Table 1 and illustrated in Figs 1–5. Additionally, significant mean and median differences, respectively, will be reported in the following sections.

### VPP variables

The range of the VPP median height was between 0.13 m in handstand and 0.49 m in backwards walking, as shown in Table 1. The median VPPx value differed in each condition maximal by ±0.01 m from the horizontal CoM position. The VPP position is exemplarily illustrated in Fig 1, the median values of all participants are shown in Fig 2A and 2B. The median R^2^ value was high in all conditions, ranging from R^2^ = 0.93 for backwards walking to R^2^ = 0.99 for walking with shoes. The R^2^ value is shown in Fig 2C and 2D.

**Table 1:** Statistical analysis of VPP and spatio-temporal parameters. Virtual pivot point (VPP) data are median±median absolute deviation between participants and mean over all trials. The horizontal (x) and vertical (z) position of the VPP are calculated for R^2^>0.6. Spatio-temporal data are mean±s.d. between participants and mean over all trials. The speed is calculated as mean value of the contact. Additionally, contact time, single support phase (SSP) time and double support phase (DSP) time, step length, step width, and cadence are shown. “rel” denotes the relative duration of the phase with respect to the contact time. Significant P-values (P<0.05) are in bold. Post hoc analysis with Šidák correction revealed significant differences between conditions: differences from barefoot, shoes and plank are indicated with ‘a’, ‘b’ and ‘c’, respectively (P<0.05).

	barefoot	shoes	backwards	handstand	plank	stilts	P-value	F-value/*η*^2^
**VPP variables**								
R^2^	0.98±0.01	0.99±0.01	0.93±0.02	0.97±0.74	0.98±0.01	0.98±0.00	0.325	1.07/0.10
VPPx (m)	0.00±0.01	0.00±0.01	-0.01±0.01	-0.01±0.02	0.00±0.01	0.01±0.01	**0.014**	5.33/0.35
VPPz (m)	0.32±0.08	0.26±0.07	0.49±0.10	0.13±0.11	0.43±0.07^*b*^	0.33±0.08	**0.000**	11.96/0.55
**spatio-temporal**								
speed (m s^−1^)	0.61±0.07	0.62±0.08	0.53±0.06^*a*^	0.43±0.07^*a*^	0.61±0.06	0.56±0.04^*b*, *c*^	**0.000**	25.33/0.72
contact time (s)	0.97±0.04	0.98±0.05	0.99±0.06	1.06±0.17	0.99±0.05	0.95±0.06	**0.146**	2.37/0.19
SSP time (s)	0.50±0.03	0.51±0.04	0.52±0.06	0.32±0.06^*a*^	0.49±0.04	0.54±0.03^*c*^	**0.000**	79.82/0.89
DSP time (s)	0.23±0.02	0.24±0.02	0.24±0.02	0.37±0.07^*a*^	0.25±0.02	0.21±0.01^*b*, *c*^	**0.000**	33.15/0.77
rel. SSP (%)	51.95±3.25	51.79±2.98	52.19±4.11	30.38±4.82^*a*^	49.62±2.76^*b*^	56.25±2.37^*b*, *c*^	**0.000**	112.04/0.92
step length (m)	0.43±0.04	0.44±0.05	0.38±0.03^*a*^	0.29±0.04^*a*^	0.44±0.04	0.40±0.02^*b*, *c*^	**0.000**	37.64/0.79
step width (sm)	0.24±0.03	0.25±0.04	0.32±0.03^*a*^	0.44±0.04^*a*^	0.25±0.02	0.27±0.03	**0.000**	139.56/0.93
cadence (steps min^−1^)	85.92±5.17	84.02±5.72	86.97±6.34	92.51±11.22	83.93±6.52	84.74±4.73	0.075	3.19/0.24

The VPPz was significantly higher in plank walking than in walking with shoes (P<0.001). In all conditions, the VPPz was significantly higher than the CoM (P<0.030). No significant differences were found in the VPPx position for the considered comparisons. In backwards walking, the VPPx was significantly more posterior than the CoM (P = 0.006). In all other conditions, no significant differences between the VPPx and the CoM position were found. For the R^2^, no significant differences between the conditions could be observed. Only one participant showed an R^2^ value smaller than 0.6 (at handstand walking, R^2^ = -32.74, illustrated in Fig 3B). All other R^2^ values were greater than 0.8 for all participants and conditions, as illustrated in Fig 2D.

### VPP related variables

No differences in spatio-temporal variables (Table 1) and time profile of GRFs and CoM (Fig 4A–4D) could be observed between barefoot and shoe walking and thus, no significant differences in VPP variables could be found. The absolute CoP position was more anterior in the shoe condition than in the barefoot condition, especially in late stance (Fig 4F).

Walking backwards was slower than walking barefoot with shorter and wider steps (P<0.001). The slower speed was due to the shorter steps and the controlled cadence. The horizontal GRFs showed less propulsion while walking backwards compared to walking barefoot. The vertical GRFs had a slightly higher braking peak and a lower propulsion peak when walking backwards (Fig 4A and 4B). The horizontal CoM position has smaller range for walking backwards. While the CoP (relative to the horizontal CoM position) was more posterior in the backwards condition than in the barefoot condition, the CoP (absolute) was slightly more anterior in the single support phase and more posterior in late stance. In the double support phase of backwards walking, even a posterior movement could be observed. The more posterior CoP (relative) position also shifts the VPPx more posterior. The behavior of the input variables compensate each other such that apart from that no other effects on the VPP variables could be observed.

Handstand walking was slower than barefoot walking, with shorter and wider steps, and a shorter single support phase for handstand (P<0.001). All events of the horizontal GRFs profile appeared earlier for the handstand walking than for barefoot walking. In handstand, the braking peak was higher and the propulsion peak lower. The mean vertical GRFs showed lower (more precisely: no) braking and propulsion peaks for the handstand condition as compared to the barefoot condition. The range of the horizontal CoM was considerably smaller for handstand walking. The CoP (relative to the horizontal CoM position) was more posterior in the handstand condition than in the barefoot condition until late stance. In the double support phase, it shifts more anterior. Until the end of the single support phase, the CoP (absolute) moved only slightly forward (~2 cm), then it shifted strongly anteriorly (~8 cm) in the double support phase. The CoP (absolute) is always more posterior in handstand walking than in barefoot walking. The GRFs compensate each other so that no effect on the VPP could be observed. The CoP shifts the VPPx more posterior, the lower CoMz shifts the VPPz down in handstand walking compared to barefoot walking, but without statistical effect.

For the plank condition, the relative single support phase was shorter than for the shoe condition (P = 0.012). For the horizontal GRFs, lower peaks could be observed at the plank condition, especially at propulsion. This shifts the VPPz upwards. The CoP (absolute and relative to the horizontal CoM position) was in late stance, i.e. double support phase, more anterior for plank.

Walking with stilts was slower, with shorter steps, and with a longer single support phase than for shoe walking (P<0.041). The horizontal GRF peaks were higher for stilt walking. The horizontal CoM showed a lower range of motion for the stilt condition. While the CoP (relative to the horizontal CoM position) had a larger range of motion for stilt walking, the CoP (absolute) was more posterior and with lower range than for shoe walking because of the reduced contact area. The higher GRFx peaks would shift the VPPz down, the larger relative CoP range would shift the VPPz up. These effects seem to compensate each other.

For the comparison of stilts and plank, the same effects could be observed (P<0.041 for spatio-temporal comparisons). They are even stronger when looking at the peaks of the horizontal GRFs.

## Discussion

### Effect of manipulating the CoP on VPP

All analyzed conditions were appropriate for manipulating the CoP behavior and thus the foot rollover movement, as illustrated in Fig 4F. However, a VPP could nevertheless be observed in all conditions, with only a single exception for the handstand walking of one participant (Fig 3B). In all conditions, the horizontal VPP position differs only slightly or not at all from the horizontal CoM position, while the vertical VPP position is significant above the CoM.

Therefore, the first part of hypothesis (1), that the VPP could be observed for walking with modified foot rollover, can be confirmed. The second part of hypothesis (1) which assumed that the VPP position will differ with changed foot rollover can not be confirmed at this point, presumably because of the large variance between participants and trials. Only for the comparison of shoe walking and plank walking could a significant difference in VPP height be observed. However, the direction of the difference is contrary to the assumption. This is presumably due to the relatively small influence of the isolated CoP movement on the VPP, especially since the VPP is calculated for the single support phase while the changes of the CoP predominantly occur at the double support phase (Fig 4E and 4F). This is also reflected in the profile of the ankle torque, where no obvious differences between shoe and plank walking could be found (Fig 5B). The shift of the vertical VPP position can be attributed to the shorter single support phase and the lower GRFx peaks in plank walking compared with shoe walking (Table 1 and Fig 4A), since the GRFx is an important variable for the VPP position [21]. That means that the changed CoP movement only influences the VPP position indirectly.

**Fig 6:**
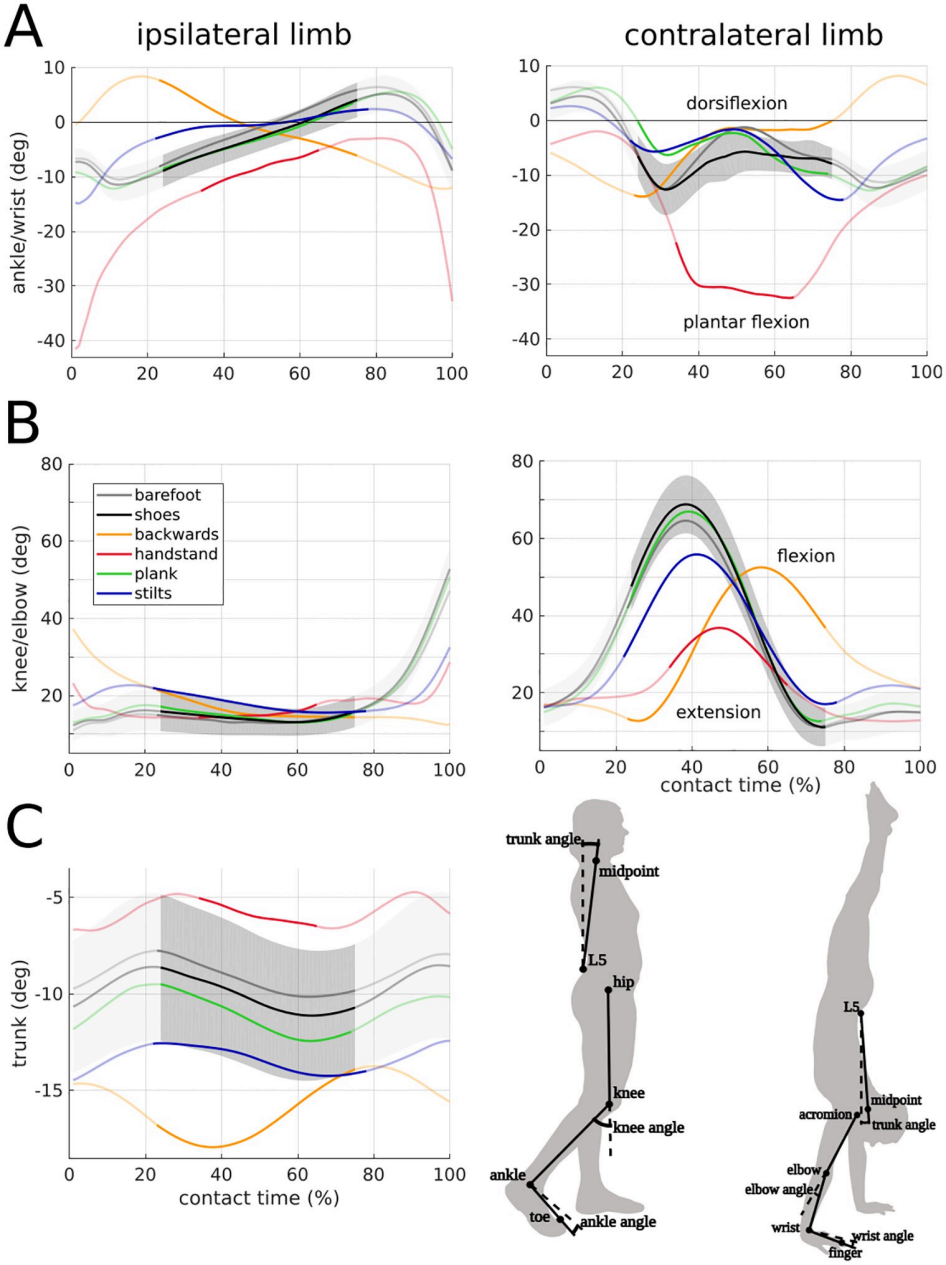
Joint angles for ipsi- and contralateral limbs. All experimental conditions are shown. Values are mean of all trials and subsequent mean of all participants (N = 11). For shoe walking, mean±s.d. (gray area) is shown. The non-transparent trajectory represents the single support phase, for which the VPP was calculated. A: Ankle angle (for handstand walking wrist angle), B: knee angle (for handstand walking elbow angle), and C: trunk angle are shown. The trunk angle is calculated as the angle between the vertical and the linear connection of the midpoint between both acromion markers and L5.

Hypothesis (2) that handstand and backwards walking show a lower R^2^ has to be rejected. However, although the values are not statistically significant, a larger scatter and tendency for lower R^2^ values for these conditions can be observed (Fig 2D). Additionally, handstand and backwards walking show the largest deviations from barefoot walking, as the baseline condition, in the profile of the joint angles (Fig 6) and the ankle/wrist torque (Fig 5B) can be observed, compared to the other conditions.

The VPP variables (position and R^2^) confirm former studies, especially for the baseline conditions barefoot and shoe walking [1, 2, 8, 9, 21]. Until now, all human gait experiments regarding the perturbation of the VPP could show the point, except for single outliers [4, 8, 9, 21]. Thus it may be concluded that the VPP has a fundamental function for upright human gait. However, it has to be emphasized that the definition of the threshold of R^2^ for the existence of the VPP is rather arbitrarily chosen, based on the rating of one study [19]. Maybe here the value of 0.6 is too small which would explain why the VPP occurs in most of the experiments. On the other hand, the differences in R^2^ values between outliers and the others are large enough to assess the threshold value as acceptable.

### Has the VPP a fundamental function for upright gait?

In this study one participant showed a deviant behavior for handstand walking. The R^2^ value is lower than 0.6 (even lower than zero) for three of seven trials. While the profile of the angular momentum of this participant lies inside the first standard deviation of all participants (Fig 5A), differences in the GRFs and the duration of the contact phases could be observed. This participant showed a low braking peak and a high propulsion peak at the horizontal GRFs and thus a very unbalanced (with net propulsion) integral of GRFs. This leads to a strong propulsion in walking direction, which is also represented in the VPP plot. This plot is, for nearly all trials, not fan-like i.e., not balanced around CoM, but the force vectors are mostly oriented anterior to the CoM, as illustrated in Fig 3B. Additionally, the zero crossing of the horizontal GRFs takes place in the first double support phase. This is due to a long first double support phase and an early zero crossing. This is contrary to all other participants where the zero crossing take place at the single support phase. The behavior of the outlier participant is interesting because here walking without a VPP could be observed in more than one trial.

In contrast to the experiments, but in analogy to the outlier, a simulation study showed stable walking without VPP was possible [22]. The horizontal GRFs were balanced around zero for the whole contact since steady-state walking was investigated. However, analogous to the outlier participant of this study, the horizontal GRFs were not balanced in the single support phase and the single support phase was shorter than for the VPP gaits. This simulation was based on the muscle-reflex-model of Geyer & Herr [23], where the foot is modeled as rigid block, similar to the plank condition in this study. That means that the foot rollover differs from human walking, but nevertheless a VPP emerges for the original model of Geyer & Herr. The primary VPP model of Maus et al. [1] only has spring legs with points as feet, analogous to the stilts of this study. This comparison between models and experiments suggests that changes in foot rollover do not generate considerable changes in VPP. Rather it seems that only the entire interaction of the musculoskeletal system and its perturbation affect the existence of the VPP. This perturbation could be found for the non-VPP gait of the simulation study [22].

One limitation in this study is that only the single support phase was considered when calculating the VPP. To make a more general statement, the whole contact should be investigated, especially for the outlier trials without VPP. Secondly, only one contact was analyzed. To get a more smooth, steady-state gait with a longer cycle, it would be better to use a treadmill instead of force plates. If the different gait types of this study were possible on a treadmill, this would also improve the performance, since the participants do not have to concentrate on hitting the force places exactly or hold the speed given by the metronome. Thirdly, only young and healthy participants were investigated, for older or diseased people the results could vary.

### Future considerations

In all previous experiments it is assumed that the input variables of the VPP interact in the way that one compensates perturbations of other variables. Only for single outliers (e.g. this study and [21]) the VPP input variables seem not to be able to compensate each other. Following from that it would be interesting to find perturbations of the entire interaction of the musculoskeletal system. Here, neurological disorders could be analyzed concerning the VPP. In a former study, no differences between healthy controls and patients with Down syndrome could be observed for VPP height and R^2^, but only for the anterior-posterior VPP position [24]. However, it is possible that the deviation from a non-pathological gait pattern is not sufficient large for patients with Down syndrome concerning the existence of the VPP. Therefore, patients with neurological disorders showing prominent changes of gait and thus of the entire interaction of the musculoskeletal system, like Parkinson’s disease, spasticity, or stroke should be further analyzed. In a study concerning post-stroke participants, a changed angular impulse compared with neurotypical individuals was observed, and so it was supposed that no VPP occurs [25]. This could be confirmed by future studies. For this issue, the authors have developed a VPP calculation tool for an easy opportunity to calculate the VPP. This tool can be found at [26] and the community is invited to take their own data and find a gait without VPP with the help of the VPP calculation tool.

The next step after finding out whether a VPP exists or not is to find the reason for its (non-) existence. In former studies, it was assumed that the VPP is necessary for postural stability [1] or, in contrast, to increase locomotion efficiency [22]. In studies with exoskeletons it was found that if the control is based on the VPP model, walking is more energy-efficient, i.e. the metabolic costs are reduced [27, 28]. This supports the suggestion that for human walking without an exoskeleton the VPP is used for energy reasons. Since it is assumed that the energy needs increase for some neurological disorders as mentioned above, it would be interesting to conclude the energy component in the VPP analysis. However, it is laborious to realize the measurement of the required energy amount for human gait. Here, further studies could measure the metabolic costs and VPP variables of human gait or find proper models to estimate these costs. The knowledge could also be used to optimize the energy efficiency of walking with ortheses and protheses, or humanoid robots, due to the application of the VPP.

## Abbreviations

BW: body weight
CoM: center of mass of the whole body
CoP: center of pressure
DSP: double support phase
GRFs: ground reaction forces
R^2^: coefficient of determination
SSP: single support phase
TD: touchdown
TO: take-off
VPP: virtual pivot point
x: horizontal, anterior-posterior
z: vertical
